# Gene expression profile indicates involvement of uniconazole in *Coix lachryma‐jobi* L. seedlings at low temperature

**DOI:** 10.1002/fsn3.1338

**Published:** 2019-12-16

**Authors:** Yulan Huang, Caijun Yue, Junliang Xiang, Yiqiang Han, Jingwei Wang, Liyan Wang, Lifang Sun

**Affiliations:** ^1^ College of Life Science and Technology Heilongjiang Bayi Agricultural University Daqing China; ^2^ College of Agronomy Heilongjiang Bayi Agricultural University Daqing China

**Keywords:** coix seedlings, growth and physiological parameters, low temperature, transcriptome, uniconazole (UNZ)

## Abstract

Uniconazole (UNZ) can alleviate a variety of abiotic stresses such as low temperature. With application of UNZ on *Coix lachryma‐jobi* L. (coix) under low‐temperature stress, growth and physiological parameters were investigated in seedlings. Meanwhile, transcriptome profile in coix seedlings was characterized as well. The results showed an increase of 11.90%, 13.59%, and 10.98% in stem diameter, the aboveground and belowground biomass in 5 mg/L uniconazole application group (U3), compared with control check low‐temperature group (CKL). Some anti‐oxidase activities also show significant difference between CKL and U3 (*p* < .05). Transcriptome results showed that 3,901 and 1,040 genes had different expression level at control check (CK) and CKL, CKL and U3. A considerable number of different expressing genes (DEGs) related to the plant hormone signal transduction, photosynthesis, reactive oxygen species (ROS)‐related genes, and secondary metabolism in response to uniconazole application were identified in this study. The transcriptomic gene expression profiles present a valuable genomic tool to improve studying the molecular mechanisms underlying low‐temperature tolerance in coix. At the same time, it would provide a certain basis for the application of UNZ in the production of coix resistance under low temperature.

## Graphical abstract

With application of S3307 on *Coix lachryma‐jobi* L (coix) under low‐temperature stress, growth and physiological parameters were investigated in seedlings. Meanwhile, using Illumina sequencing technology, transcriptome profile in coix seedlings was characterized as well.

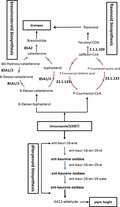

## 1 INTRODUCTION

Coix, commonly known as Job's tears, is an annual crop that has long been consumed as both a herbal medicine and a nourishing food (Lin & Li, 2016). Coix is a nourishing food containing 16.2% proteins, 4.65% lipids, and 79.17% carbohydrates (Kim et al., 2004). Modern scientific studies demonstrate that adlay seeds have exhibited antitumor (Lu, Zhang, Jia, Wu, & Lu, 2011), anti‐inflammatory (Chen, Chung, Chiang, & Lin, 2011), and anti‐allergic properties (Chen, Shih, Hsu, & Chiang, 2010). With the increasing value of coix, the research on coix has drawn more and more attention.

Temperature greatly affects the growth and development of crops. When crops are exposed to low‐temperature stress, the growth and production may be suppressed in some crops (Li & Colin, 2011; Li et al., 2017). At present, coixs mainly grow in south China including Hubei, Yunnan, Guizhou, Guangdong, and Guangxi in south China. There is fewer coix planting in north China including Liaoning, Jilin, and Heilongjiang, which are located on the Northeast plain and belong to the cold zone. The effective accumulated temperature is insufficient, and cold damage frequently occurs in Heilongjiang province (Chen, Tian, et al., 2014; Chen, Song, et al., 2014). The low temperature seriously affects the germination, emergence, growth, and yield of coix.

It has been well documented that plant growth regulators (PGRs) play important roles in crop production and resisting environmental stresses (Prat, Botti, & Fichet, 2008). As a member of the triazole PGRs family, UNZ was increasingly applied in crops (Wu, Sun, Zhang, & Liu, 2013). This chemical could regulate numerous growth and development processes, such as flowering period (Cao, Zhang, Zhang, & Jiang, 2003), increasing output and quality (Liu et al., 2016), and enhancing plant resistance (Zhang et al., 2007). Some studies showed that UNZ exerted beneficial effects on alleviating adverse stresses (Gan, He, Yan, & Hu, 2012; Wu et al., 2013; Zhen et al., 2012). For example, UNZ could improve the anti‐oxidation system of the omega plant under low‐temperature stress, enhance the photosynthetic electron transfer efficiency in plants, and reduce the damage to leaf cells under low temperature (Zheng et al., 2016). When sorghum treated by uniconazole, the SOD, POD, and CAT activities showed a trend of earlier increasing and later decreasing with UNZ concentration increasing (Liang, Guo, Zhou, Zhang, & Yan, 2016). High‐throughput sequence analysis is an efficient and powerful method for transcriptome analysis, and over the past several years, the technology has increasingly been used to characterize transcriptomic in plants. It was particularly useful in nonmodel species, whose genomic sequences were often unavailable, such as black cottonwood, *Prunus dulcis* Mill, *Beta vulgaris*, *Eucalyptus dunnii*, and *Chrysanthemum nankingense* (Liu, Jiang, Lan, Zou, & Gao, 2014; Moliterni et al., 2015; Mousavi et al., 2014; Ren et al., 2014; Tang et al., 2015). Previous studies mainly focused on the effect of UNZ on the aspects of physiology and biochemistry under adverse stress. However, the effect of UNZ on the transcriptome under low‐temperature stress in coix has not been investigated yet.

In this work, we studied the physiological parameters and relevant transcriptome of coix in response to UNZ under low‐temperature stress. The DEGs generated by de novo assembly were annotated and analyzed according to unigene's GO annotation and KEGG metabolic pathways. Thus, this study aimed to find the correlation between the physiological parameters and DEGs via the transcriptome analysis, which would be useful for the application of UNZ in the production of coix. At the same time, it provided some references for the coix cultivation in Heilongjiang and other cold regions.

## 2 MATERIAL AND METHODS

### 2.1 Plant materials and UNZ treatments

The experiments were conducted in an environmental controlled room at Agricultural Department of Heilongjiang Bayi Agricultural University. Coix seeds were sterilized with 10% NaClO solution for 30 min, rinsed thoroughly three times with distilled water, and germinated at 28°C in the dark. After 96 hr, germinated seedlings were transferred to Hoagland's nutrient solution, in which seedlings were grown in a greenhouse at 25–30°C with a 13‐hr/11‐hr (day/night) photoperiod and 60%–80% humidity. The nutrient solution was renewed every other day, and the pH was maintained at 5.8. When two leaves of plant were fully expanded, seedlings were subjected to treatments (Huang, Xiang, & Yin, 2017). A wide range of concentrations of UNZ was examined in preliminary tests. The adequate range of UNZ solution was from 0 to 15 mg/L (Mei, Zheng, Wang, & Li, 2014). Specific experimental designs were shown in Table S1, and each treatment consists of three replications. According to growth and physiological parameters measurement, 5 mg/L (U3) of UNZ in coating agent was selected as the most appropriate concentration for experiment.

### 2.2 Growth and physiological parameters measurement

After 72 hr of the low temperature, thirty plants per treatment were harvested for the measurement of plant height, root length, and stem diameter measured with vernier caliper in minimum value 0.02 mm. The aboveground and belowground samples of coix seedlings were oven‐dried at 105°C for 30 min and then dried at 65°C for 72 hr. The dry biomass of aboveground and belowground were recorded and calculated by average.

The fresh leaf materials taken from different treatments were extracted with 1:1 alcohol and acetone mixture. The leaf chlorophyll a and b concentrations in the supernatant of the solution were measured using a spectrophotometer at 663 and 645 nm, respectively (Strain & Svec, 1966).

Method for obtaining enzyme solution was as follows: Enzymes were extracted from 0.5 g leaves or roots with a prechilled mortar being added 0.1 M phosphate buffer (pH 7.8) and were centrifuged at 15,000 *g* for 15 min. The supernatants were used to assay enzyme activity, in which operations for the enzyme extractions preparation were performed at 4°C (Maia, Costa de Macedo, Voigt, Freitas, & Silveira, 2010). Superoxide dismutase (SOD) activity was determined by nitroblue tetrazolium (Karanlik, 2001). Peroxidase (POD) activity was determined according to the method of Cakmak, Strbac, and Marschenr (1993). Catalase (CAT) activity was assayed and calculated by monitoring the initial rate of H_2_O_2_ disappearance at 240 nm (Gong, Zhu, Chen, Wang, & Zhang, 2005). Ascorbate peroxidase (APX) activity was assayed and calculated by the changes in absorbance at 290 nm (Nakano & Asada, 1981).

### 2.3 Extraction, testing of RNA and cDNA library construction, and sequencing

Mixed samples of five leaves in the treatment of CK, CKL, and U3 were used for RNA extraction (Invitrogen Trizol Reagent of RNA extraction kit 15596018). A cDNA library was constructed using a NEB kit for library preparation and sequenced using an Illumina HiSeq2500 (Beijing Biomarker Technologies Co.)

### 2.4 The transcriptome assembly and genes functional annotation

High‐quality sequencing reads data were first broken into short fragments (K‐mer) using Trinity software, and then, the short fragments were extended to longer fragments (Contig) (Grabherr et al., 2011). The fragment set (Component) was obtained through overlapping these fragments. Lastly, transcription sequence was identified in each fragment set using the method of De Bruijn (Grabherr et al., 2011; Haas et al., 2013).

Unigene sequences were compared in the following databases using BLAST software (http://blast.ncbi.nlm.nih.gov/Blast.cgi) to obtain annotation information for unigenes. Based on BLAST parameters, those unigenes with an E‐value less than 10^–5^ were selected. Sequences of selected unigenes were aligned within databases (Nr, COG, Swiss‐Prot, KEGG, GO).

### 2.5 Calculating unigenes expression lever and detecting differentially expressed genes

Sequencing reads of each sample were compared with the unigene database using the Bowtie method (Langmead, Trapnell, Pop, & Salzberg, 2009) and then processed with RSEM (Li, Wang, Zhang, & Wang, 2011) to estimate the expression levels with the FPKM value.

DEGs sets for CK, CKL, and U3 were acquired by differential expression analysis using EBSeq (Leng, Dawson, Thomson, Ruotti, & Rissman, 2013). During this screening process, FDR < 0.01 and fold change (FC) ≥ 2 were used as screening criteria. The selected DEGs were then hierarchically clustered (Murtagh & Legendre, 2014).

### 2.6 Enrichment analysis of differentially expressed genes

The DEGs screened were mainly analyzed by GO function and KEGG pathway enrichment. We extracted GO annotations of DEGs, mapped GO function to the corresponding secondary features based on unigene's GO annotation (Ye et al., 2018), and drew the histogram. The KEGG pathway enrichment analysis was implemented via KOBAS2.0 (http://kobas.cbi.pku.edu.cn/home.do) (Xie, Mao, Huang, & Wei, 2011).

### 2.7 Quantitative real‐time PCR verification

According to RNA‐Seq data, some specific genes were selected for the analysis of their expressions by the RT‐PCR (reverse transcription polymerase chain reaction). Primers were designed using Gene Runner software (Hastings Software, New York, USA). The sequences and the annealing temperature of each primer are shown in the Table S4. Total RNA (1 μg) from each selected gene was treated with DNAse I (Invitrogen), translated into first‐strand cDNA, which was synthesized with TransScript One‐Step gDNA Removal and cDNA Synthesis SuperMix (TransGene Biotech), and then stored at −20°C for subsequent analysis. Each PCR reaction contained 20 μl mixture, consisting of 2 μl cDNA, 10 μl of 2× TransStart Top Green qPCR SuperMix, and 0.4 μl of the forward and reverse primers. All qRT‐PCRs were performed in three technical replicates in Bio‐Rad CFX96 and performed in two steps: predenaturation for 2 min at 94°C, 45 cycles of denaturation for 2 s at 94°C, and annealing/extension for 15 s at 60°C.

After amplification, the PCR products were sequenced to check the specificity of the primer sets (Table S4). Outliers were manually discarded, and the housekeeping gene actin was used as internal standard to calculate the relative expression level, which were standardized to the transcript levels for *PtACTIN* calculated by the 2^−ΔΔCt^ method (Livak & Schmittgen, 2001).

### 2.8 Statistical Analysis

All the data presented in the tables and figures were the mean values of three replicates at least, in which results were expressed as mean ± standard deviation (*SD*). The data were subjected to statistical analysis by SPSS 20.0 for testing the significance of differences.

## 3 RESULTS

### 3.1 Effects of S3307 on growth and physiological parameters of coix seedlings under low‐temperature stress

The plant height, root length, stem thickness, and aboveground and belowground biomass under CKL showed a significant decreasing trend compared with CK group. The UNZ treatment group could increase stem thickness, biomass, and lateral root numbers and reduce plant height and root length to a certain extent. Comparing to the CKL, the plant stem diameter was increased by 11.90%, lateral root numbers increased by 31.04%, and the aboveground and belowground biomass increased by 13.59% and 10.98%, respectively (Table 1), while plant height and root length were decreased by 6.62% and 7.95%, respectively.

**Table 1:** Effects of exogenous UNZ on the growth of coix seedlings under drought stress

Treatment	Plant height /cm/plant	Stem diameter /mm/plant	Root length /cm/plant	Aboveground biomass /mg/10 plants	Belowground biomass /mg/10 plants	Lateral root number
CK	27.74 ± 0.28a	0.42 ± 0.03a	20.46 ± 0.61a	1.10 ± 0.01a	0.93 ± 0.03a	4.00 ± 0.58ab
CKL	26.40 ± 0.42ab	0.37 ± 0.01c	18.23 ± 0.40b	0.89 ± 0.01c	0.81 ± 0.01b	3.33 ± 0.33b
U1	25.97 ± 0.44bc	0.39 ± 0.03ab	18.11 ± 0.38b	0.89 ± 0.01c	0.82 ± 0.01b	4.33 ± 033ab
U2	25.48 ± 0.11bcd	0.40 ± 0.01ab	16.82 ± 0.13c	0.97 ± 0.02b	0.90 ± 0.01a	4.67 ± 0.33ab
U3	24.54 ± 0.23cde	0.42 ± 0.02ab	16.78 ± 0.19c	1.03 ± 0.04bc	0.91 ± 0.01a	5.33 ± 0.33a
U4	24.28 ± 0.65de	0.41 ± 0.02ab	16.52 ± 0.29c	1.03 ± 0.04bc	0.90 ± 0.01a	5.33 ± 0.33a
U5	23.86 ± 0.78e	0.40 ± 0.08ab	16.43 ± 0.57c	0.99 ± 0.03b	0.90 ± 0.01a	5.00 ± 0.58a

Note

Mean ± *SD* (*n* = 3) are shown in table. Same letter indicates no significant difference between treatments (*p* < .05). CKL: low‐temperature group; U1, U2, U3, U4, and U5: 1, 3, 5, 7, and 9 mg/L UNZ concentration.

**Figure 1:**
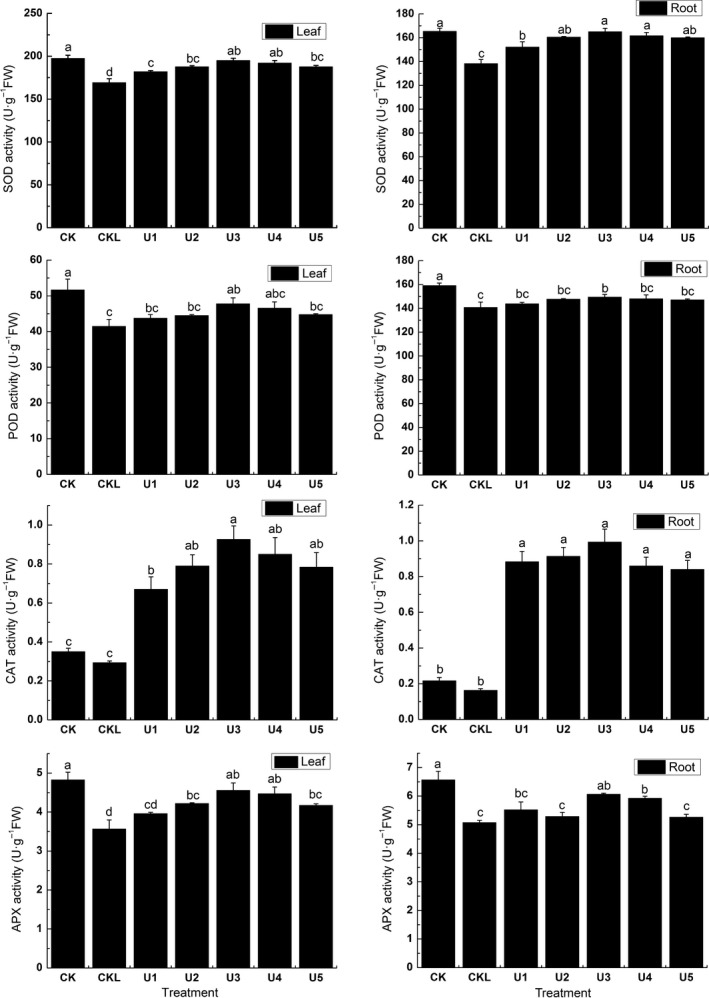
Effects of different concentration UNZ on the anti‐oxidant enzymes activities of coix seedlings under low‐temperature stress

Low‐temperature stress decreased POD, CAT, APX, and SOD activities significantly (*p* < .05). After uniconazole, the activities of CAT, SOD, and POD of leaves in U3 increased by 13.22%, 13.19%, 68.41%, and 21.71% comparing to CKL (*p* < .05). The SOD, POD, and CAT activities of the roots increased by 16.19%, 5.79%, 83.83%, and 16.34% comparing to CKL, respectively (*p* < .05), from Figure 1. With the increasing of the uniconazole concentration, the anti‐oxidant enzymes activities showed the trend of first increasing then decreasing within the scope of the experimental concentration. Final results showed that 5 mg/L uniconazole could remarkably improve anti‐oxidant enzymatic activities of coix seedling under low‐temperature stress.

**Figure 2:**
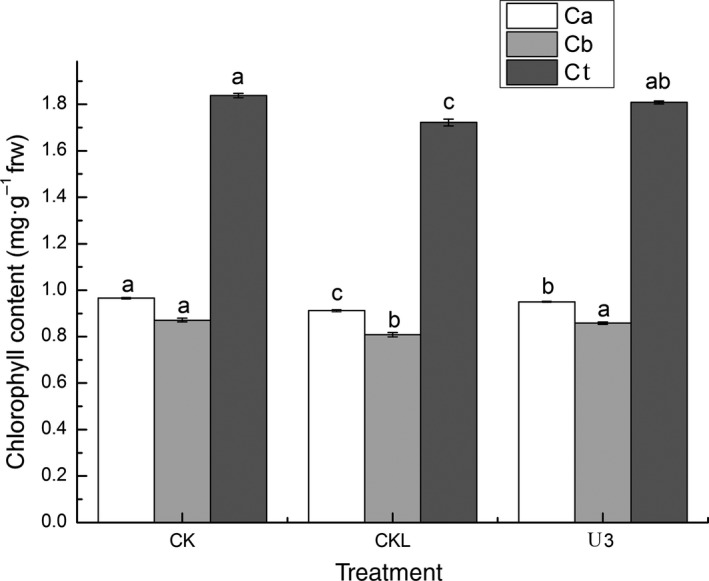
Effects of UNZ on chlorophyll concentration of coix seedlings under low‐temperature stress

Chlorophyll a, chlorophyll b, and total chlorophyll decreased significantly after low‐temperature stress from Figure 2 (*p* < .05). The chlorophyll a content of the U3 group increased significantly compared with CKL (*p* < .05), while was not significant compared with CK (*p > *.05). The chlorophyll b and total content of the U3 group increased significantly compared with CKL and CK (*p* < .05). The chlorophyll b and total content of the U3 group was 6.10% and 5.01% higher than that of the CKL group separately.

### 3.2 RNA‐seq and transcriptome profiles of UNZ in response to low‐temperature stress for coix leaves

To comprehensively investigate the transcriptome and gene expression profiles of coix seedlings under normal condition, low‐temperature stress, and UNZ treatment, three cDNA samples from coix leaves were prepared and sequenced using Illumina HiSeq x‐ten platform. An overview of the RNA‐Seq reads derived from the three libraries was presented in Table S2. After the low‐quality reads were removed, 21.16 Gb clean reads were obtained with an average of 6.99 Gb reads for each sample, and the proportion of Q30 was greater than 91.37%, which indicated that the sequencing results were highly accurate.

With the Trinity program (Grabherr et al., 2011), a total of 246,201 transcripts were obtained from the clean reads with an N50 length of 2,555 bp and a mean length of 1,590.62 bp. Among them, 82,274 unigenes were generated with an average length of 763.45 bp. The length distributions of transcripts and unigenes were given in Table S3, which suggested that the sequencing assembly was ideal.

### 3.3 Gene annotation and functional classification

To predict and analyze the function of the unigenes, we carried out functional annotation by using BLAST against multiple databases such as Nr, Swiss‐Prot, GO, COG, KOG, KEGG, eggNOG, and Pfam. Of the 82,274 unigenes, 35,103 (42.66%) unigenes were successfully matched to homologous sequences in at least one of databases. Among them, 8,268 (10.04%), 24,986 (30.36%), 10,459 (12.71%), 16,078 (19.54%), 22,047 (26.80%), 19,975 (24.28%), 31,467 (38.24%), and 34,496 (41.93%) unigenes were found in COG, GO, KEGG, KOG, Pfam, Swiss‐Prot, eggNOG, and Nr databases, respectively. The Nr database produced the largest number of annotations. Compared with other species, coix showed the most matches to *Sorghum bicolor* (14,815), followed by *Zea mays* (9,391) and *Setaria italica* (2,230) (Figure S1).

GO assignments system was used to classify the possible functions of coix genes. A total of 24,986 unigenes were successfully annotated and classified into 50 functional groups of three major GO categories (cell component [CC], molecular function [MF], and biological processes [BP]) (Figure S2). The top three GO terms for classified genes were cell (14,372), cell part (14,347), and organelle (11,296) for cell component category; catalytic activity (12,982), binding (13,661), and transporter activity (1,856) for molecular function; and metabolic process (13,324) and cellular process (12,813) for biological processes, which were related with low‐temperature stress.

To further analyze the unigenes, we searched the annotated sequences for the genes involving in COGs classifications. All unigenes were aligned to COG database to predict and classify possible functions, and COG function classification of consensus sequences was showed in Figure S3. Among these classification, the largest group was general function prediction (973), followed by carbohydrate transport and metabolism (836), signal transduction mechanisms (819), secondary metabolites biosynthesis, transport and catabolism (752), translation, ribosomal structure, and biogenesis (712).

### 3.4 Differentially expressed gene and KEGG enrichment analysis among three samples

**Figure 3:**
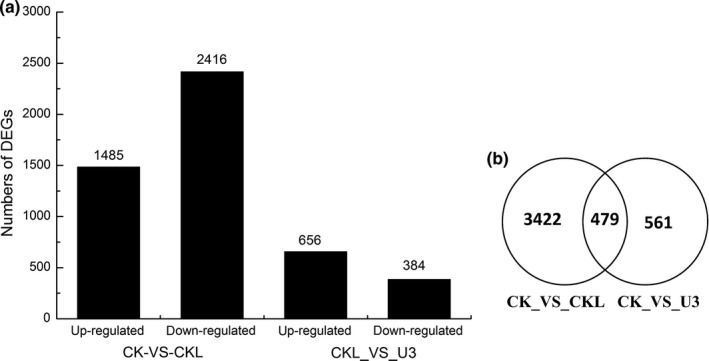
(a) Summary of differentially expressed genes in different treatment. Bar graph indicating the DEGs among the sets of CK_VS_CKL and CKL_VS_U3 (a). (b) Up‐regulated and down‐regulated DEG number of groups in different treatment group (b)

Wilcoxon signed‐rank test (FDR < 0.01 and fold change ≥ 2) showed 3,901 and 1,040 genes had different expression level at CK_VS_CKL and CKL_VS_U3, respectively (Figure 3a). In particular, there were 1,485 up‐regulated and 2,416 down‐regulated DEGs at CK_VS_CKL and 656 up‐regulated and 384 down‐regulated at CKL_VS_U3. Relatively, the number of down‐regulated DEGs was far more than that of up‐regulated under CK_VS_CKL, while the number of up‐regulated DEGs was far more than that of down‐regulated under CKL_VS_U3 (Figure 3a,b). These results indicated that the expression levels of numerous different genes in coix seedlings were affected by the low temperature and uniconazole.

The significantly enriched pathways of DEGs under CK_VS_CKL and CKL_VS_U3 were represented as Table S5. Under CK_VS_CKL, 632 DEGs were assigned to the KEGG database involving 116 pathways. Under CKL_VS_U3, 168 DEGs were assigned to 88 pathways.

As shown in Table S5, the major pathways involved in the cold response were photosynthesis, flavonoid biosynthesis, phenylpropanoid biosynthesis, porphyrin and chlorophyll metabolism, plant hormone signal transduction, carbon fixation in photosynthetic organisms, starch and sucrose metabolism, and glutathione metabolism in CK_VS_CKL. The major pathways involving the UNZ response were phenylpropanoid biosynthesis, starch and sucrose metabolism, glutathione metabolism, diterpenoid biosynthesis, tropane, piperidine and pyridine alkaloid biosynthesis, brassinosteroid biosynthesis, glyoxylate, and dicarboxylate metabolism, which were related to secondary metabolism pathway.

### 3.5 Differential expression of plant hormone signal transduction‐related genes

Approximately 43 DEGs involving plant hormone signal transduction pathways, such as 5 bZIP transcription factor (*bZIP*), 4 AUX/IAA family (*AUX/IAA*), 3 auxin response factor (*auxin*), 1 jasmonic acid‐amido synthetase JAR2 (*JAR2*), 1 ethylene receptor 3 isoform X3 (*Ethylene*), putative GID1‐like gibberellin receptor (*Gibberellin*), and BR‐signaling kinase 2 (*BR*), were found between CK and CKL. Among these genes, up‐regulated genes were more than down‐regulated genes. There were 7 annotated DEGs in plant hormone signal transduction pathways between CKL and U3. Among them, AUX/IAA and auxin were significantly up‐regulated. These results suggested that the expression level change in many genes should be directly associated with the dynamic characteristics of UNZ regulating the plant hormone signal transduction of coix responding to low temperature (Table 2).

**Table 2:** Different regulating patterns CK_VS_CKL and CKL_VS_U3 for DEGs associated with plant hormone signal transduction‐related genes

Gene ID of maize genome database	Gene annotation	CK_VS_CKL		CKL_VS_U3	
		Log 2 FC	*p* value	Log 2 FC	*p* value
c48342.graph_c0	(bZIP) bZIP transcription factor TRAB1	2.915817	*p* < .01	–	*–*
c19663.graph_c0	(bZIP) bZIP transcription factor 23	3.970731	*p* < .01	–	*–*
c21254.graph_c0	(bZIP) bZIP transcription factor TRAB1	−2.46691	*p* < .01	–	*–*
c44103.graph_c1	(bZIP) bZIP transcription factor 23	3.580574	*p* < .01	–	*–*
c21955.graph_c0	(AUX/IAA) Auxin‐responsive protein IAA17	−3.09981	*p* < .01	–	*–*
c35574.graph_c0	(AUX/IAA) IAA9—auxin‐responsive Aux/IAA family member	4.692104	*p* < .01	–	*–*
c21290.graph_c1	(AUX/IAA) Auxin‐responsive protein IAA17	−2.56518	*p* < .01	–	*–*
c41306.graph_c2	(AUX/IAA) auxin‐responsive protein IAA14	3.237346	*p* < .01	–	*–*
c20465.graph_c0	(AUX/IAA) auxin‐responsive protein IAA2 isoform X2	–	*–*	1.341306	*p* < .01
c30004.graph_c0	(AUX/IAA) auxin‐responsive protein SAUR71	–	*–*	1.384291	*p* < .01
c17602.graph_c0	(Auxin) auxin transporter‐like protein 3	–	*–*	3.445028	*p* < .01
c47964.graph_c0	Jasmonic acid‐amido synthetase JAR2	−3.60945	*p* < .01	–	*–*
c49480.graph_c0	Ethylene receptor 3 isoform X3	3.139991	*p* < .01	–	*–*
c20776.graph_c0	Putative GID1‐like gibberellin receptor	2.589646	*p* < .01	–	*–*
c43381.graph_c0	BR‐signaling kinase 2	3.342611	*p* < .01	–	*–*

### 3.6 Differential expression of photosynthesis and Ros‐related genes

Approximately 80 DEGs involving photosynthesis and Ros pathways were identified to be different between CK and CKL, such as photosystem I reaction center subunit IV (*PsaE*), photosystem II reaction center PSB28 protein (*PSB28*), photosystem I subunit O (*PSAO*), photosynthetic NDH subunit of lumenal (*NDH*), photosystem I reaction center subunit psaK (*psaK*), photosystem II core complex proteins psbY (*psbY*), photosystem I psaG/psaK (*psaG/psaK*), photosystem I reaction center subunit N (*PsaD*), chloroplastic, chloroplastic, peroxisomal (S)‐2‐hydroxy‐acid oxidase, and peroxisomal (S)‐2‐hydroxy‐acid oxidase. Among these DEGs, most genes were down‐regulated genes. Low temperatures could decrease level of Ros in study, in which results were inconsistent with Sharma (Sharma, Shahzad, Kumar, et al., 2019; Sharma, Shahzad, Rehman, et al., 2019), due to different species possibly. Forty‐seven annotated DEGs were identified in photosynthesis and Ros pathways between CKL and U3. Among them, *chloroplastic, peroxidase 2* and *peroxidase 12*, *pyruvate, phosphate dikinase 2 isoform X1*, *peroxidase N OS = Armoracia rusticana*, *peroxisomal (S)‐2‐hydroxy‐acid oxidase GLO3*, *peroxidase 52*, *peroxidase 21*, and *peroxidase 54* were significantly up‐regulated. These results suggested that the expression level change in many genes would be directly associated with the dynamic characteristics of UNZ regulating photosynthesis and Ros pathways of coix responding to low temperature (Table 3).

**Table 3:** Different regulating patterns CK_VS_CKL and CKL_VS_U3 for DEGs associated with photosynthesis and Ros‐related genes

Gene ID of maize genome database	Gene annotation	CK_VS_CKL		CKL_VS_U3	
		Log 2 FC	*p* value	Log 2 FC	*p* value
c46603.graph_c0	(PsaE) Photosystem I reaction center subunit IV	−4.24341	*p* < .01	–	*–*
c15699.graph_c0	(PSB28) Photosystem II reaction center PSB28 protein	−3.4977	*p* < .01	–	*–*
c46598.graph_c0	(PSAO) Photosystem I subunit O	−5.0462	*p* < .01	–	*–*
c31179.graph_c0	(NDH) Photosynthetic NDH subunit of lumenal location 2	−5.69283	*p* < .01	–	*–*
c44372.graph_c1	(psaK) Photosystem I reaction center subunit psaK	−3.60104	*p* < .01	–	*–*
c15960.graph_c0	(psbY) Photosystem II core complex proteins psbY	−2.67123	*p* < .01	–	*–*
c46608.graph_c0	(psaG/psaK) Photosystem I psaG/psaK	−5.18788	*p* < .01	–	*–*
c46597.graph_c0	(PsaD) Photosystem I reaction center subunit N	−4.13528	*p* < .01	–	*–*
c39828.graph_c0	NADP‐dependent malic enzyme	–	*–*	1.199615	*p* < .01
c16053.graph_c0	Ferredoxin (2Fe‐2S) (chromatophore)	−2.83731	*p* < .01	1.375573	*p* < .01
c20566.graph_c1	Photosystem I chlorophyll a/b‐binding protein 6	–	*–*	1.122583	*p* < .01
c43324.graph_c0	Ribulose bisphosphate carboxylase small chain 3A/3C, chloroplastic	−4.13528	*p* < .01	5.31685	*p* < .01
c44460.graph_c1	Pyruvate, phosphate dikinase 2 isoform X1	–	*–*	1.28114	*p* < .01
c16053.graph_c1	Ferredoxin, root R‐B1 OS = Raphanus sativus	−2.74096	*p* < .01	1.45244	*p* < .01
c15959.graph_c0	Plastocyanin, chloroplastic	−4.28003	*p* < .01	1.195391	*p* < .01
c22272.graph_c0	Peroxisomal (S)‐2‐hydroxy‐acid oxidase	−2.42988	*p* < .01	–	*–*
c38073.graph_c0	Peroxisomal (S)‐2‐hydroxy‐acid oxidase	−2.91842	*p* < .01	–	*–*
c39930.graph_c1	Peroxidase 2 (Fragment)	–	*–*	2.825538	*p* < .01
c46393.graph_c1	Peroxidase N OS = Armoracia rusticana	–	*–*	1.968199	*p* < .01
c32884.graph_c1	Peroxidase 12	–	*–*	3.625566	*p* < .01
c35048.graph_c0	Peroxisomal (S)‐2‐hydroxy‐acid oxidase GLO3	–	*–*	1.550146	*p* < .01
c48075.graph_c0	Peroxidase 52	–	*–*	1.496224	*p* < .01
c47956.graph_c0	Peroxidase 21	–	*–*	1.101328	*p* < .01
c15832.graph_c1	Peroxidase 54	–	*–*	1.181505	*p* < .01

### 3.7 Differential expression of primary and secondary metabolism‐related genes

Approximately more than 14 DEGs were found involving in primary pathways, the majority of the DEGs were involved in amino acids and sugar metabolism in CKL and U3, such as *peroxidase 52*, *peroxidase 21*, *isocitrate lyas peroxidase N*, *peroxidase 2*, *peroxidase 12*, *tricin synthase 1*, *putrescine hydroxycinnamoyltransferase*, *peroxidase 54*, *isocitrate lyase*, *ribulose bisphosphate carboxylase small chain*, *galacturonosyltransferase*, *ornithine decarboxylase*, *alpha‐amylase*, and *beta‐amylase 1*. Some DEGs related to secondary metabolism also were annotated, such as *triacylglycerol lipase*, *achilleol B synthase*, *dihydroflavonol 4‐reductase*, and *cytochrome P450 85A1*. These results suggested that the expression change in many genes would be directly associated with the dynamic characteristics of UNZ regulating the primary and secondary metabolism of coix responding to low temperature (Table 4).

**Table 4:** Different regulating patterns CK_VS_CKL and CKL_VS_U3 for DEGs associated with primary and secondary metabolism‐related genes

Gene ID of maize genome database	Gene annotation	CK_VS_CKL		CKL_VS_U3	
		Log 2 FC	*p* value	Log 2 FC	*p* value
c48075.graph_c0	Peroxidase 52	−3.22178	*p* < .01	1.496224	*p* < .01
c47956.graph_c0	Peroxidase 21	−4.59505	*p* < .01	1.101328	*p* < .01
c47228.graph_c0	Isocitrate lyase	–	*–*	4.622478	*p* < .01
c46393.graph_c1	Peroxidase N	–	*–*	1.968199	*p* < .01
c40547.graph_c1	Cationic peroxidase SPC4	–	*–*	1.525964	*p* < .01
c39930.graph_c1	Peroxidase 2 (Fragment)	–	*–*	2.825537	*p* < .01
c39930.graph_c0	Peroxidase 12	–	*–*	2.272243	*p* < .01
c36431.graph_c1	Peroxidase 12	–	*–*	1.966595	*p* < .01
c32884.graph_c1	Peroxidase 12			6.255662	*p* < .01
c20048.graph_c0	Tricin synthase 1	3.012590	*p* < .01	2.444736	*p* < .01
c16538.graph_c0	Putrescine hydroxycinnamoyltransferase	−5.925466	*p* < .01	1.909574	*p* < .01
c15832.graph_c1	Peroxidase 54	–	*–*	1.181505	*p* < .01
c10553.graph_c0	Probable cinnamyl alcohol dehydrogenase	–	*–*	2.097522	*p* < .01
c48855.graph_c0	Triacylglycerol lipase 2	–	*–*	1.605362	*p* < .01
c47228.graph_c0	Isocitrate lyase	–	*–*	4.622478	*p* < .01
c45764.graph_c1	Isocitrate lyase	–	*–*	2.261063	*p* < .01
c43324.graph_c0	Ribulose bisphosphate carboxylase small chain 3A/3C, chloroplastic	–	*–*	5.316849	*p* < .01
c42996.graph_c1	Achilleol B synthase	–	*–*	2.509108	*p* < .01
c41036.graph_c1	Dihydroflavonol 4‐reductase	–	*–*	1.031617	*p* < .01
c40891.graph_c0	Probable galacturonosyltransferase 3	−2.776106	*p* < .01	1.354334	*p* < .01
c40804.graph_c0	Ornithine decarboxylase	−3.324458	*p* < .01	4.523741	*p* < .01
c38774.graph_c0	Alpha‐amylase	*–*	*–*	2.357640	*p* < .01
c37577.graph_c0	Beta‐amylase 1, chloroplastic	*–*	*–*	1.455868	*p* < .01
c36172.graph_c0	Alpha‐l‐arabinofuranosidase	*–*	*–*	2.860008	*p* < .01
c31968.graph_c0	Cytochrome P450 85A1	*–*	*–*	1.431418	*p* < .01

Note

*p* value showing multiple check *p* value and significant for statistical differences.

Log 2 FC showing the difference multiple of the sample quality test expression (fold change) being taken log2, which can reduce the gap between values that are particularly different and those that are relatively small.

### 3.8 Quantitative real‐time PCR (qRT‐PCR) confirmation

To confirm the accuracy of the Illumina RNA‐Seq results, 9 of the transcripts related photosynthesis, sugar, and secondary metabolism were selected for qRT‐PCR (Table S4). The expression levels of these DEGs with qRT‐PCR were compared with those of DEGs with RNA‐Seq. A significant correlation (*r* ^2^ = .97237) was observed between the RNA‐Seq and qRT‐PCR (Figure S4). The qRT‐PCR results were consistent with their transcript abundance in RNA‐seq, which verified the accuracy of the DEGs from RNA‐seq analyses in this experiment.

## 4 DISCUSSION

In this work, we explored physiochemical and transcriptomic changes of coix in response to UNZ under low‐temperature stress. Our physiochemical data clearly demonstrated that the coix seedlings experienced complex changes in growth parameters, anti‐oxidant enzymes, and chlorophyll contents (Figures 1 and 2; Table 1). These changes further suggested that UNZ could strengthen the ability to withstand low temperature by increasing stem diameter and aboveground and belowground biomass. The results were consistent with the findings (Liu et al., 2019; Mei et al., 2014; Qiu, Wang, Yan, & Jin, 2005).

**Figure 4:**
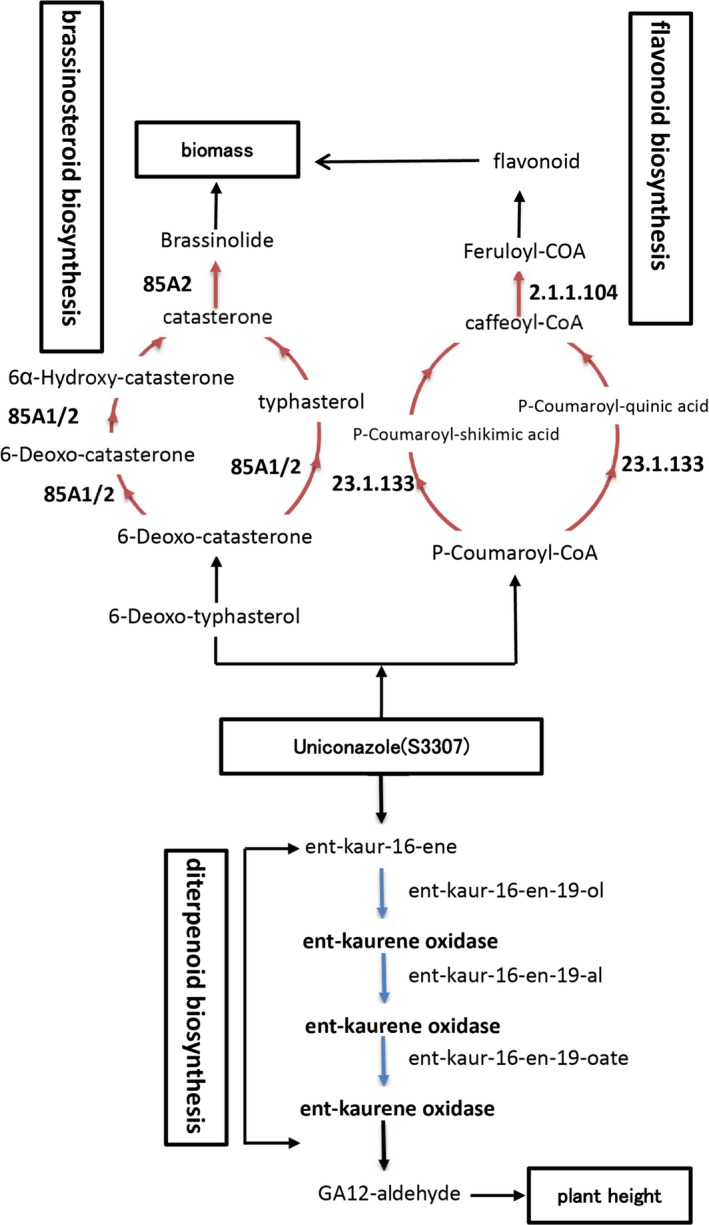
Affect of uniconazole on the diterpenoid biosynthesis, flavonoid, and brassinosteroid pathway. Red arrows indicate positive regulation, and blue arrows indicate negative regulation

In this study, the major pathways involved in the cold response were photosynthesis, flavonoid biosynthesis, phenylpropanoid biosynthesis, plant hormone signal transduction, and carbon fixation in photosynthetic organisms, as well as starch and sucrose metabolism under low temperature. The pathways were closely associated with low‐temperature stress (Chen, Tian, et al., 2014; Chen, Song, et al., 2014; Sharma, Shahzad, Kumar, et al., 2019; Sharma, Shahzad, Rehman, et al., 2019; wang et al., 2018; Xu et al., 2014). The major pathways involving in the UNZ response mechanism were phenylpropanoid biosynthesis, starch and sucrose metabolism, glutathione metabolism, diterpenoid biosynthesis, flavonoid biosynthesis, and brassinosteroid biosynthesis, which were closely associated with low‐temperature resistance (Li et al., 2016). UNZ could enhance low‐temperature tolerance in coix, which may be related with brassinosteroid biosynthesis and diterpenoid biosynthesis (Figure 4). UNZ primarily inhibited gene expression in relation to diterpenoid biosynthesis metabolic pathways. It was evident that uniconazole could down‐regulate the genes coding ent‐kaurene oxidase involving in the GA3 metabolism, which were consistent with UNZ catalyzing the oxidation of ent‐kaurene to ent‐kaurenoic acid in gibberellin biosynthesis (Izumi, Kamiya, Sakyrai, Oahio, & Takahashi 1985). UNZ treatments altered endogenous hormone content, especially for GA. Furthermore, shikimate O‐hydroxycinnamoyltransferase and caffeoyl‐CoA O‐methyltransferase related to the synthesis of flavonol were annotated and up‐regulated. Meanwhile, brassinosteroid‐6‐oxidase (CYP85A2) related to the synthesis of brassinosteroid was also annotated and up‐regulated, which can enhance dry weight of aboveground and belowground biomass of plants (Huang et al., 2016) and this phenomenon was also observed in our study (Table 1).

Plant hormones played essential roles in response to abiotic stress through their involvement in signaling networks and physiological processes (Colebrook, Thomas, Phillips, & Hedden, 2014; Lu et al., 2014; Pan et al., 2016; Pantin et al., 2013). In this study, some functional gene coding hormones were significantly affected. These hormones include auxin, jasmonic acid (JA), gibberellin (GA), brassinolide (BR), and their signal components could participate in UNZ‐mediated plant defense processes. In Figure 4, adding uniconazole could inhibit the genes coding ent‐kaurene oxidase, which were involved in the GA3 metabolism and the major oxidase gene families. GA12 was regulated in the coix seedling system which confirmed the feedback mechanism of GA content. The major oxidase gene of GA families was all detected after uniconazole treatment (Han et al., 2017). The 13S‐lipoxygenase coded jasmonic acid synthesis was also up‐regulated. Auxin was an essential morphogenetic signal participating in the regulation of cell identity during plants development, and auxin signaling pathways constituted a critical component of mechanisms for plant tolerance to abiotic stresses (Pan et al., 2016). Substantial evidence has demonstrated a tight link between auxin responses and cold stress (Jain & Khurana, 2009; Rahman, 2013). In this study, auxin‐responsive protein IAA2 isoform X2, auxin‐responsive protein SAUR7 (c30004.graph_c0), and auxin transporter‐like protein 3 (c17602.graph_c0) were significantly up‐regulated under UNZ (Table 2). Therefore, it was reasonable to speculate that auxin intervenes in coix seedling in response to low‐temperature stress. The study demonstrated the complexity of hormone interaction in coix seedlings responding to low‐temperature stress after UNZ treatment.

## 5 CONCLUSION

Our dataset included comprehensive analysis in sequence and DEGs profiling data that provide a dynamic perspective on transcriptomic variations in coix seedlings responding to UNZ under low temperature. Many DEGs were simultaneously found in CK‐VS‐CKL and CKL‐VS‐U3 libraries in different functional pathways, such as plant hormone signaling, photosynthesis, and Ros, as well as secondary metabolism. These results provided novel insights into the mechanism of UNZ enhancing the tolerance of coix seedlings to low‐temperature stress with respective of physiology and transcriptome. At the same time, it provided some references for the coix cultivation in Heilongjiang and other cold regions.

## CONFLICT OF INTEREST

The authors declare that they do not have any conflict of interest.

## ETHICAL APPROVAL

This study does not involve any human or animal testing.

## Supporting information

FigS1

FigS2

FigS3

FigS4

TableS1‐S5
