# Supplementary figures and images for: Gene expression profile indicates involvement of uniconazole in Coix lachryma‐jobi L. seedlings at low temperature
Source: Food Sci Nutr. 2019 Dec 16;8(1):534–46. doi: 10.1002/fsn3.1338 (PMC6977508; doi:10.1002/fsn3.1338)

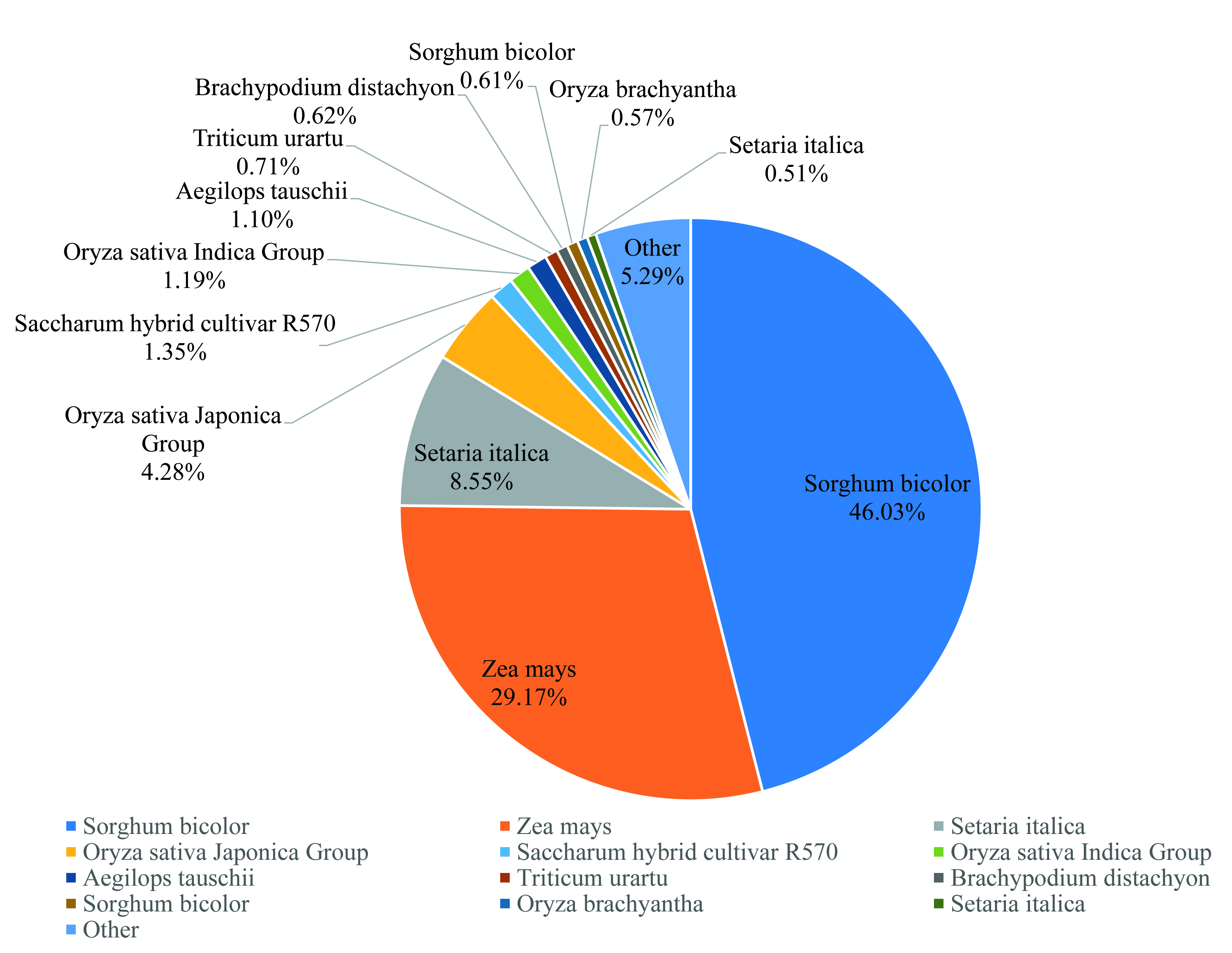

Supplement: Supplementary file 1 — FigS1 [file FSN3-8-534-s001.tif]

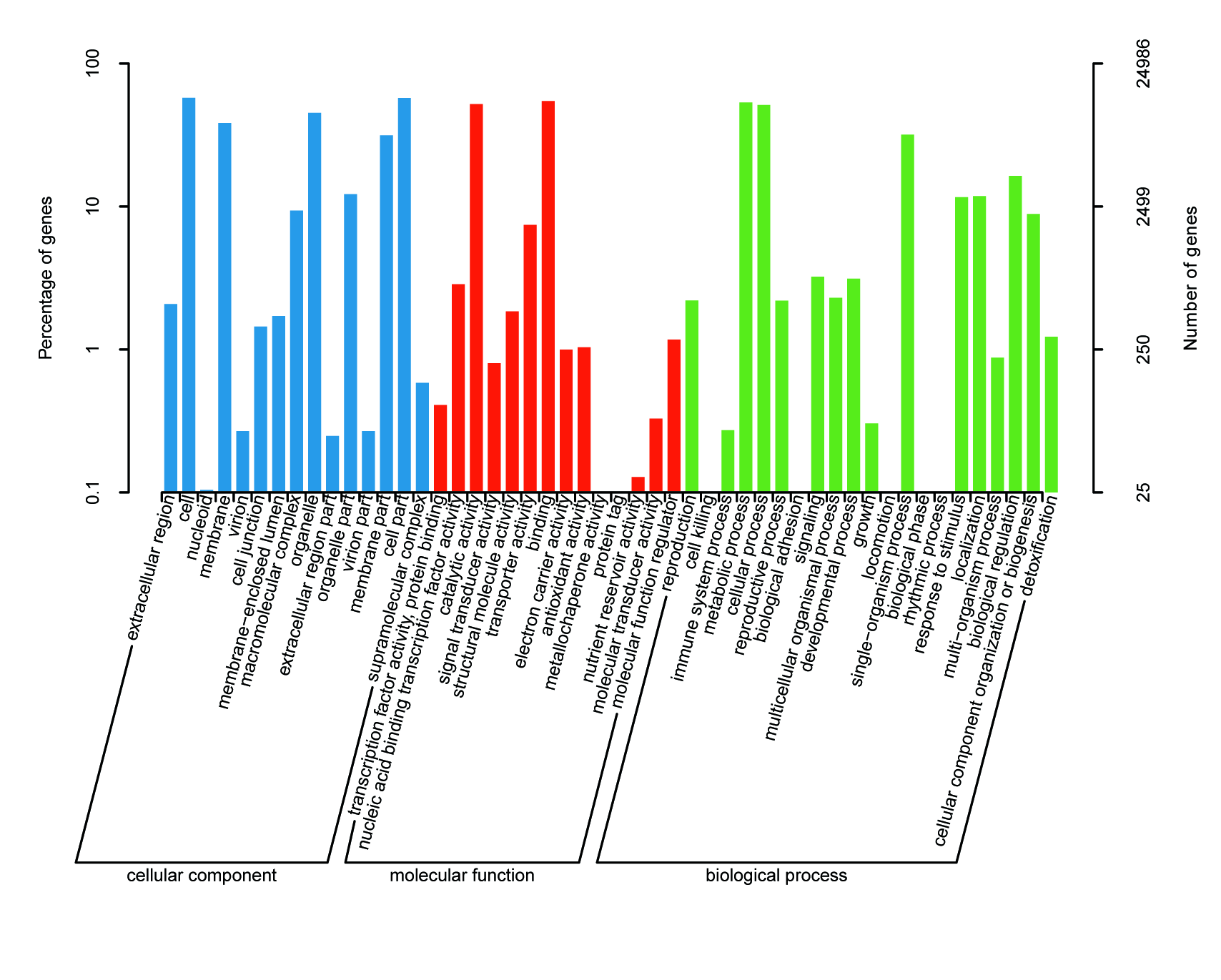

Supplement: Supplementary file 2 — FigS2 [file FSN3-8-534-s002.tif]

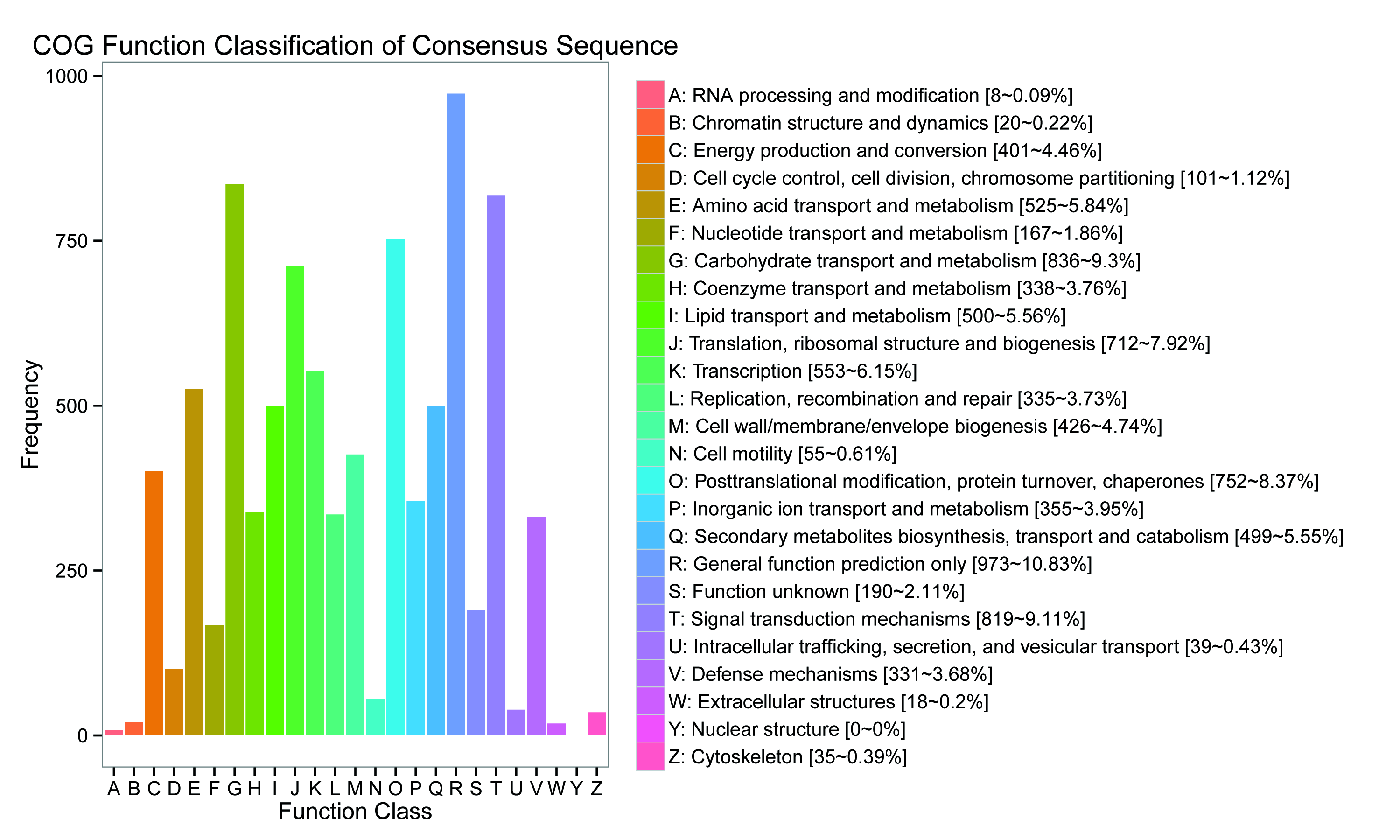

Supplement: Supplementary file 3 — FigS3 [file FSN3-8-534-s003.tif]

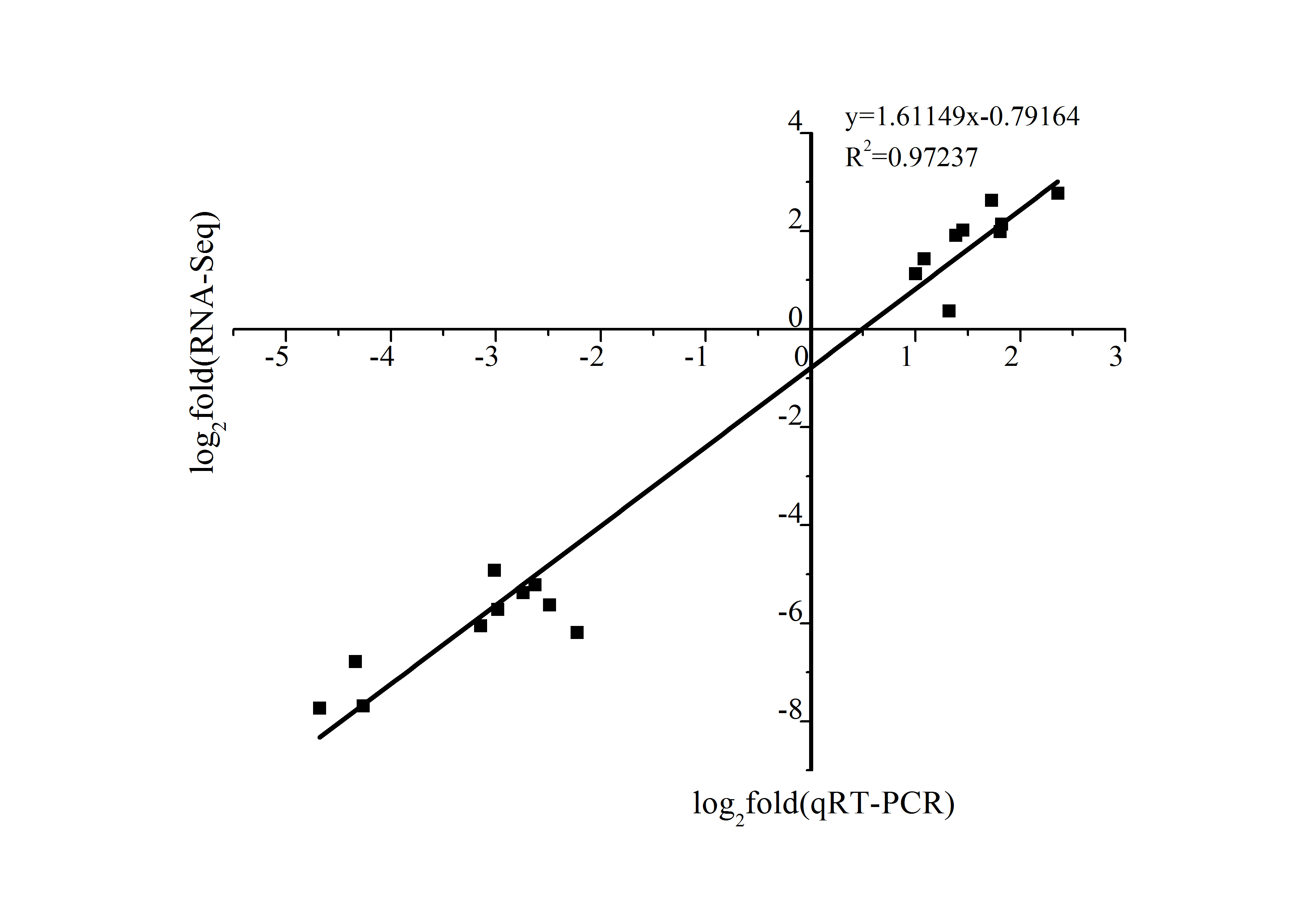

Supplement: Supplementary file 4 — FigS4 [file FSN3-8-534-s004.tif]
