# Supplementary material for: Gene expression profile indicates involvement of uniconazole in Coix lachryma‐jobi L. seedlings at low temperature
Source: Food Sci Nutr. 2019 Dec 16;8(1):534–46. doi: 10.1002/fsn3.1338 (PMC6977508; doi:10.1002/fsn3.1338)
Supplement: Supplementary file 5 — TableS1‐S5 [file FSN3-8-534-s005.doc]

**Table S1** Test design

| Treatment | Temperature/℃ | UNZ/ mg·L^-1^ |
| --- | --- | --- |
| CK | 25 | 0 |
| CKL | 5 | 0 |
| U1 | 5 | 1 |
| U2 | 5 | 3 |
| U3 | 5 | 5 |
| U4 | 5 | 7 |
| U5 | 5 | 9 |

**Table S2** Summary for raw reads of three samples

| Samples | Read Number | Clean Data | GC Content | %≥Q30 |
| --- | --- | --- | --- | --- |
| CK | 23,395,663 | 6,992,597,648 | 59.68% | 91.37% |
| CKL | 23,703,791 | 7,091,649,396 | 56.85% | 91.43% |
| U3 | 23,663,247 | 7,077,633,718 | 56.98% | 91.89% |

Note: GC content: Clean Data G and C percentage of the total bases; Q30:Quality Score of base is greater than or equal to 30% of the total bases.

**Table S3** Summary of Illumina transcriptome assembly for *coix*

| Length Range | Transcript | Unigene |
| --- | --- | --- |
| 00-300 | 37,321(15.16%) | 29,146(35.43%) |
| 300-500 | 32,552(13.22%) | 20,590(25.03%) |
| 500-1000 | 41,555(16.88%) | 15,365(18.68%) |
| 1000-2000 | 59,607(24.21%) | 9,894(12.03%) |
| 2000+ | 75,166(30.53%) | 7,279(8.85%) |
| Total Number | 246,201 | 82,274 |
| Total Length | 391,612,029 | 62,811,934 |
| N50 Length | 2,555 | 1,388 |
| Mean Length | 1590.62 | 763.45 |

Note: 246,201 transcripts were obtained. The total length was 391,612,029 Mb, the average length was 1590.62 bp, and the N50 length was 2,555.

**Table S4** Selected genes and primers for quantitative qRT-PCR

| Gene ID | Gene | Annotation | Primer sequence 5’-3’ | Tm |
| --- | --- | --- | --- | --- |
| c16053.graph_c0 | 2Fe-2S | Ferredoxin (2Fe-2S) (chromatophore) | F:ATGAGCTTCACCTTGTAGACG  R:TTTCGCATCCCCTGTTCTC | 54.3  54.5 |
| c16194.graph_c0 | TSJT1 | stem-specific protein，asparagine synthase activity | F:ACAAGTCCACAAACTCCCTG  R:CCAAGGGCGTTAGAGTAGAAG | 53.7  52.9 |
| c18237.graph_c0 | GER5 | oxalate decarboxylase activity，Germin-like protein 8-14 | F:GTACCCGTGCAAGTCCAG  R:GTTGATTTTGCCCATGCCG | 54.5  53.5 |
| c20824.graph_c0 | ERD15 | Protein EARLY RESPONSIVE TO DEHYDRATION 15 | F:GTTGTAGGCTGTACTCTCACTG  R:TTCGCAACGGATAAGATCGG | 52.9  52.8 |
| c31002.graph_c0 | AK-2 | Probable adenylate kinase 2 | F:GAGCTGGACACCATTTCTG  R:AAAGGTTAAGTTGAGGCTGC | 53.6  53.3 |
| c39741.graph_c0 | NPF6.3 | Protein NRT1/ PTR FAMILY 6.3 | F:CGCTCTACAGTCACTACAAGTC  R:GGAAATGGATGCTCAAACGTG | 54.6  54.4 |
| c40235.graph_c0 | HAD | haloacid dehalogenase-like hydrolase | F:GATGTTCCTCTCGCTGTCG  R:TGCTTCGAGACCATGAACC | 55.6  55.1 |
| c43259.graph_c2 | MYB48 | transcription factor MYB48 | F:TTCTGGAACGACATCGCG  R:GATCCTCCAGAGCGAGTAATC | 54.3  54.9 |
| c46629.graph_c0 | TPRP-F1 | 36.4 kDa proline-rich protein precursor [Zea mays] | F:TCTTAAACACTAGAAGCGGCC  R:GTGATATACATGGAGCTGTGG | 53.7  53.4 |
| c46843.graph_c0 | At1g80440 | F-box/kelch-repeat protein At1g80440 | F:TAGTGAAGTATCAATGGCAG  R:GACAGAGTGTAGCAATTTCAGC | 52.6  52.8 |
| Action |  |  | F:CTGAACCTTTCTGACCCAAT  R:ACCTCACCGACCACCTAATG | 52.7  53.4 |

**Table S5** The most reliable top ten significantly enriched pathways of DEGs under different treatments 2.8×10^-9^

|  | Pathway ID | Pathway | DEGs in pathway | All genes  in pathway | P-value |
| --- | --- | --- | --- | --- | --- |
| CK_VS_CKL | ko00195 | Photosynthesis | 33 | 98 | 2.80×10^-9^ |
|  | ko00941 | Flavonoid biosynthesis | 23 | 60 | 4.75×10^-8^ |
|  | ko00940 | Phenylpropanoid biosynthesis | 60 | 277 | 2.95×10^-7^ |
|  | ko00860 | Porphyrin and chlorophyll metabolism | 16 | 57 | 0.000406 |
|  | ko00906 | Carotenoid biosynthesis | 11 | 36 | 0.001516 |
|  | ko04075 | Plant hormone signal transduction | 43 | 252 | 0.003462 |
|  | ko01200 | Carbon metabolism | 58 | 377 | 0.008072 |
|  | ko00710 | Carbon fixation in photosynthetic organisms | 24 | 129 | 0.009224 |
|  | ko00500 | Starch and sucrose metabolism | 42 | 261 | 0.010868 |
|  | ko00480 | Glutathione metabolism | 23 | 134 | 0.026452 |
| CKL_VS_U3 | ko00940 | Phenylpropanoid biosynthesis | 17 | 277 | 0.003824237 |
|  | ko00500 | Starch and sucrose metabolism | 16 | 261 | 0.005042813 |
|  | ko00480 | Glutathione metabolism | 10 | 134 | 0.006856338 |
|  | ko00904 | Diterpenoid biosynthesis | 4 | 26 | 0.007065222 |
|  | ko00520 | Amino sugar and nucleotide sugar metabolism | 11 | 166 | 0.011087747 |
|  | ko00960 | Tropane, piperidine and pyridine alkaloid biosynthesis | 4 | 30 | 0.011799187 |
|  | ko00905 | Brassinosteroid biosynthesis | 3 | 18 | 0.015690636 |
|  | ko00630 | Glyoxylate and dicarboxylate metabolism | 9 | 131 | 0.016795842 |
|  | ko00909 | Sesquiterpenoid and triterpenoid biosynthesis | 2 | 10 | 0.03460692 |
|  | ko00402 | Benzoxazinoid biosynthesis | 2 | 10 | 0.03460692 |
|  | ko00950 | Isoquinoline alkaloid biosynthesis | 4 | 43 | 0.039360287 |

\
